# High degree of conservancy among secreted salivary gland proteins from two geographically distant *Phlebotomus duboscqi *sandflies populations (Mali and Kenya)

**DOI:** 10.1186/1471-2164-7-226

**Published:** 2006-09-04

**Authors:** Hirotomo Kato, Jennifer M Anderson, Shaden Kamhawi, Fabiano Oliveira, Phillip G Lawyer, Van My Pham, Constance Souko Sangare, Sibiry Samake, Ibrahim Sissoko, Mark Garfield, Lucie Sigutova, Petr Volf, Seydou Doumbia, Jesus G Valenzuela

**Affiliations:** 1 Vector Molecular Biology Unit, Laboratory of Malaria and Vector Research, National Institute of Allergy and Infectious Diseases, National Institutes of Health, Rockville, Maryland, USA; 2 Department of Veterinary Hygiene, Faculty of Agriculture, Yamaguchi University, Yamaguchi, Japan; 3 Laboratory of Parasitic Diseases, National Institute of Allergy and Infectious Diseases, National Institutes of Health, Bethesda, Maryland, USA; 4 Centro de pesquisa Goncalo Moniz, Fundacao OswaldoCruz, and Faculdade de Medicina, Universidade Federal da Bahia, Salvador, Bahia, Brazil; 5 Faculty of Science and Technology, University of Bamako, Bamako, Mali; 6 Malaria Research and Training Center, Faculty of Medicine, University of Bamako, Bamako, Mali; 7 Research Technology Branch, National Institute of Allergy and Infectious Diseases, National Institutes of Health, Rockville, Maryland, USA; 8 Department of Parasitology, Charles University, Prague, Czech Republic

## Abstract

**Background:**

Salivary proteins from sandflies are potential targets for exploitation as vaccines to control *Leishmania *infection; in this work we tested the hypothesis that salivary proteins from geographically distant *Phlebotomus duboscqi *sandfly populations are highly divergent due to the pressure exerted by the host immune response. Salivary gland cDNA libraries were prepared from wild-caught *P. duboscqi *from Mali and recently colonised flies of the same species from Kenya.

**Results:**

Transcriptome and proteome analysis resulted in the identification of the most abundant salivary gland-secreted proteins. Orthologues of these salivary proteins were identified by phylogenetic tree analysis. Moreover, comparative analysis between the orthologues of these two different populations resulted in a high level of protein identity, including the predicted MHC class II T-cell epitopes from all these salivary proteins.

**Conclusion:**

These data refute the hypothesis that salivary proteins from geographically distinct populations of the same Phlebotomus sandfly species are highly divergent. They also suggest the potential for using the same species-specific components in a potential vector saliva-based vaccine.

## Background

Leishmaniasis is a vector-borne disease transmitted by Phlebotomine sandflies. The Leishmania parasite develops to an infective form inside the gut of the sandfly and is injected together with saliva into a mammalian host during blood feeding. Components present in sandfly saliva, as well as in the saliva of other arthropod vectors, have been shown to contain potent anti-hemostatic and immunomodulatory activities [1], and are able to enhance Leishmania infection [2]. Salivary proteins therefore are potential candidates for vaccines to control vector-borne diseases.

Immune responses to either sandfly salivary gland homogenate [3,4] or to the bites of sandflies [5] have been shown to protect animals against Leishmania infection. Two molecules isolated from the saliva of sandflies have been shown to confer this protection, one named "maxadilan" is a vasodilatory and immunomodulatory molecule present in the saliva of *Lutzomyia longipalpis *[6-8], and the other called PpSP15, is a molecule present in the saliva of *Phlebotomus papatasi *[9]. Maxadilan injected together with parasites was shown to enhance *Leishmania major *infection in laboratory animals as compared to injection with *Leishmania major *alone, and vaccination with maxadilan reversed this effect and protected animals against *L. major *infection [7]. Animals vaccinated with PpSP15 salivary protein developed a strong delayed-type hypersensitivity response to this protein that was sufficient to protect them against *L. major *infection since B-cell deficient animals vaccinated with PpSP15 were also protected [9].

The sand fly *Phlebotomus duboscqi *is a proven vector of *L. major *in Sub-Saharan Africa from Ethiopia to Senegal. It belongs to the subgenus Phlebotomus together with *P. papatas, P. bergeroti and P. salehii*. Electrophoretic profiles of salivary proteins of *P. duboscqi *eastern populations (Ethiopia) differ from western ones (Senegal) [10]. Cutaneous leishmaniasis has been reported in Northwest and Northeast Mali and *P. duboscqi *was reported as the suspected vector [11]. Until now there has been no information available concerning the repertoire of salivary proteins from this vector of disease, and the degree of intraspecific homogeneity present in the salivary proteins of conspecific specimens from two different geographic locations. In was previously reported that the salivary protein maxadilan, from the *Lutzomyia longipalpis *sand fly, was highly variable, up to 23% differences in amino acid identity between different sandfly populations of sandfly colonies derived from Brazil, Colombia and Costa Rica [12]. It was hypothesised that this variability was due to antigenic polymorphism that ultimately would avoid the host immune response and therefore neutralisation of a salivary protein important in blood feeding [12]. In this work we studied the salivary gland transcriptomes of *P. duboscqi *from two different locations, Mali and Kenya to test the hypothesis that sandfly salivary proteins from two geographically distinct but conspecific populations are very divergent due to the immune pressure exerted by the mammalian host. Moreover, the degree of similarity in the salivary proteins from a sand fly species originating from two different geographic locations was never investigated. Knowledge of the latter is an important aspect of vaccine development, where target proteins should exhibit a degree of conservancy within the species across its distribution range to be viable vaccine candidates.

## Results and discussion

### Sequencing of two salivary gland cDNA libraries collected in East and West Africa

**Figure 1:**
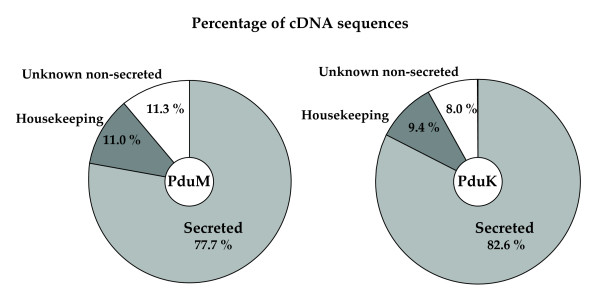
Proportion of transcripts sequenced from *Phlebotomus duboscqi *salivary gland cDNA libraries from Mali (PduM) and Kenya (PduK) populations.

We constructed and sequenced two salivary gland cDNA libraries from *P. duboscqi *collected in West Africa (Mali) and East Africa (Kenya). The total number of high-quality sequences analysed from the Mali cDNA library was 988 and from the Kenya cDNA library the sequence total was 924. The majority of the analysed transcripts from these two cDNA libraries code for secreted proteins (Figure 1). *P. duboscqi *Mali cDNA library (PduM) resulted in 77.7 % of transcripts coding for secreted proteins, 11% coding for housekeeping genes and 11.3 % coding for proteins with no clear signal secretory peptide and with unknown function (Figure 1A). Similarly, *P. duboscqi *Kenya cDNA library (PduK) resulted in 82.6% of transcripts coding for secreted proteins, 9.4 % coding for housekeeping genes and 8.0% coding for proteins with no clear signal secretory peptide and with unknown function (Figure 1B). The high percentage of secreted proteins found on *P. duboscqi *salivary gland cDNA library is similar to the ones observed in cDNA libraries from other sandflies and mosquitoes [13,14].

Table 1 and Table 2 list the transcripts coding for the most abundant and secreted salivary gland proteins from *P. duboscqi *collected in Mali and Kenya, respectively. The tables were arranged from the most abundant to the least abundant transcripts found in the two cDNA libraries. For example, transcript PduM02 is listed first and it contains 182 sequences (Table 1). The nomenclature for the transcripts on these cDNA libraries is the following: Pdu = *Phlebotomus duboscqi*, M = Mali, K = Kenya, and the number (ie: 02) denotes the contig number on the cDNA library where a contig is a cluster of identical transcripts. Many of the isolated transcripts code for proteins previously identified from the saliva of *P. papatasi *or *L. longipalpis *including PpSP15-like protein, yellow-related proteins, apyrase-like, and PpSP32-like, among others. Notably, we identified a transcript coding for adenosine deaminase (PduM73), which was previously identified in the sandfly *L. longipalpis *[15] and the mosquito *Aedes aegypti *[16] but never reported in Phlebotomus sandflies. There were also other transcripts coding for proteins not previously reported in sandflies (Table 1).

### Proteome analysis of salivary proteins from P. duboscqi from Mali and Kenya

**Figure 2:**
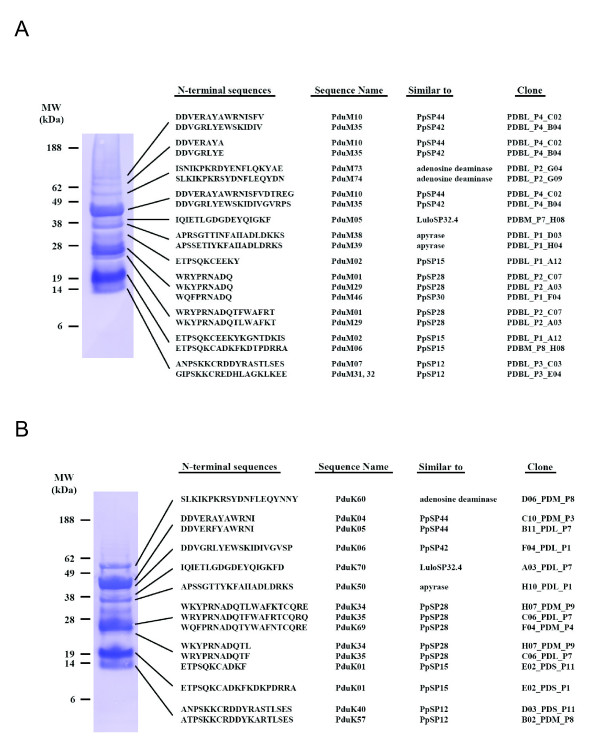
Proteome analysis of salivary gland homogenate of *Phlebotomus duboscqi *Mali (A) and Kenya (B). Salivary glands were separated as described in the methods section; N-terminal sequence was obtained and the corresponding transcript was identified utilizing salivary transcriptome information.

Edman degradation of the salivary proteins isolated from *P. duboscqi *Mali strain resulted in the identification of 16 different N-terminal sequences (Figure 2A). The transcripts coding for these N-terminal sequences were identified by searching the open reading frames of the transcripts from the constructed *P. duboscqi *cDNA database (Figure 2A). The identified proteins were two PpSP12-like proteins (PduM07, PduM31), two PpSP15-like protein (PduM02 and PduM06), three D7-related protein (PduM29, PduM01, PduM46), two apyrase-like proteins (PduM39 and PduM38), a 32-kDa protein from *L. longipalpis *(PduM05), two yellow-related proteins (PduM35, PduM10) and two adenosine deaminase-like proteins (PduM74 and PduM73). We also found proteins with the same N-terminal sequence but with different gel mobilities (PduM10, PduM35, PduM01, PduM02). These may represent the same protein with different post-translational modifications.

From *P. duboscqi *Kenya salivary gland protein analysis we found 15 different Nterminus sequences (Figure 2B). The transcripts coding for these n-terminal sequences were identified by searching the *P. duboscqi *Kenya cDNA database (Figure 2B). The identified proteins were: two PpSP12-like proteins (PduK40, PduK57), one PpSP15-like protein (PduK01), four D7-related proteins (PduK35, PduK34 and PduK69), one apyrase-like protein (PduK50), a 32-kDa protein (PduK50), three yellow-related proteins (Pduk06, PduK05 and PduK04) and an adenosine deaminase-like protein (PduK60).

### Phylogenetic tree analysis, multiple sequence alignment and identification of potential MHC class II T-cell epitopes of P. duboscqi salivary proteins

To evaluate the phylogenetic relationship among these salivary proteins and provide a better assessment of the homology of secreted salivary proteins from these two different sandfly populations, we performed sequence alignment and phylogenetic tree analysis of the most abundant and secreted proteins from the Mali and Kenya cDNA libraries. The objective of this analysis was to identify and compare the orthologues between these two geographically distant sandfly species. Because cellular immune responses to salivary proteins, particularly CD4 T cell-dependent response, are associated with protection against *Leishmania *infection, we searched for putative MHC class II T cell epitopes in these salivary proteins and compared how conserved these epitopes were between the salivary orthologues of the two sandfly populations.

Following is a description of the analysis of these salivary proteins:

### PpSP14-like proteins

**Figure 3:**
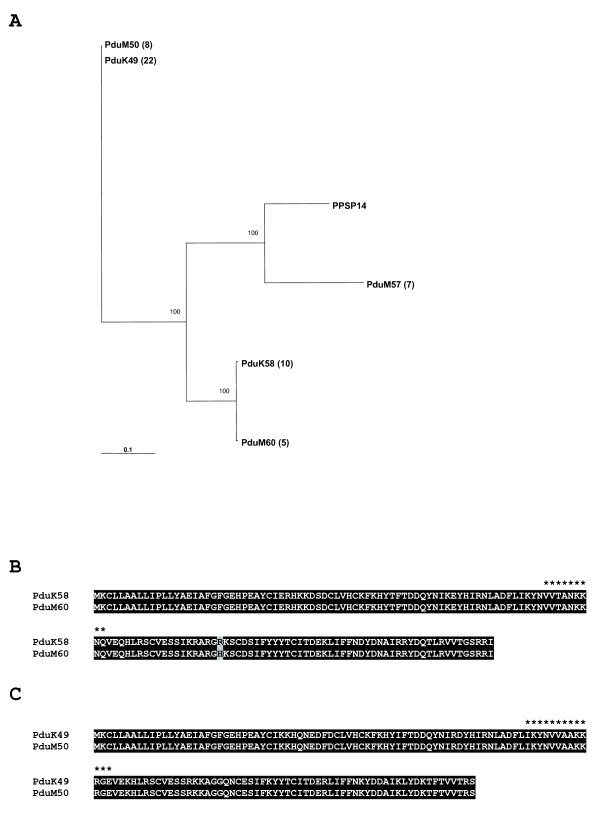
(A) Phylogenetic tree analysis of SP14-like proteins from *P. papatasi *(PpSP14), *P. duboscqi *Mali (PduM50, Pdum57 and PduM60) and *P. duboscqi *Kenya (PduK49 and PduK58). (B) Sequence alignment of the orthologues PduK58 and PduM60. Black-shaded amino acids (aa) represent identical aa, grey-shaded aa represent conserved aa and * at the top of the aa denotes potential T cell epitopes as searched by using the TEPITOPE software. (C) Sequence alignment of the orthologues PduK49 and PduM50. Black-shaded aa represent identical aa, grey-shaded aa represent conserved aa and * at the top of aa denotes potential T-cell epitopes as searched by using the TEPITOPE software.

PpSP14-like proteins are related to the 14-kDa salivary proteins of unknown function from *P. papatasi *and also identified in other sandflies, but not in other insects [9]. We found three members of this family of proteins (PduM50, PduM57 and PduM60) on the cDNA library of *P. duboscqi *Mali strain and two members (PduK49 and PduK58) in the Kenya strain. Phylogenetic tree analysis of these salivary proteins resulted in the formation of three distinct clades, one containing PduM50 and Pduk49, the second containing PpSP14 and PduM57, and the third containing PduK58 and PduM60 (Figure 3). PduM50 and PduK49 is a cluster of orthologous sequences (COG) as well as PduM60 and PduK58. When comparing the number of transcripts among these orthologues, PduK49 has 22 transcripts and PduM50 has only eight transcripts, overall, these sequences contained more (PduK49) or fewer (PduM50) sequences from a particular value than expected from a random distribution, as evaluated by the χ^2 ^test, this suggests that this transcript/protein may be more represented in the Kenya population than in the Mali population. PduK58 has ten transcripts and PduM60 has five transcripts, which may suggest that these transcripts/proteins may be represented in similar proportions in these two populations. PduM57 may be a transcript/protein present only in the Mali population or rarely in the Kenya population. Sequence comparison of these orthologues resulted in a 99.3% identity between PduM60 and PduK58 (Figure 3B) and 99.8% identity between PduM50 and PduK49 (Figure 3C). Because cellular immune responses (specifically a DTH response) to sandfly proteins are related to protection against Leishmania infection, we wanted to identify potential MHC class II-restricted epitopes in these salivary proteins and determine whether these epitopes were conserved when comparing two different sandfly populations. We identified an epitope in PduM60, VVTANKKNQ (Figure 3B), which is 100% identical in PduK58. The epitope for PduM50 and PduK49 is IKYNVVAAKKRGE (Figure 3C), which is also 100% identical.

### PpSP15-like proteins

**Figure 4:**
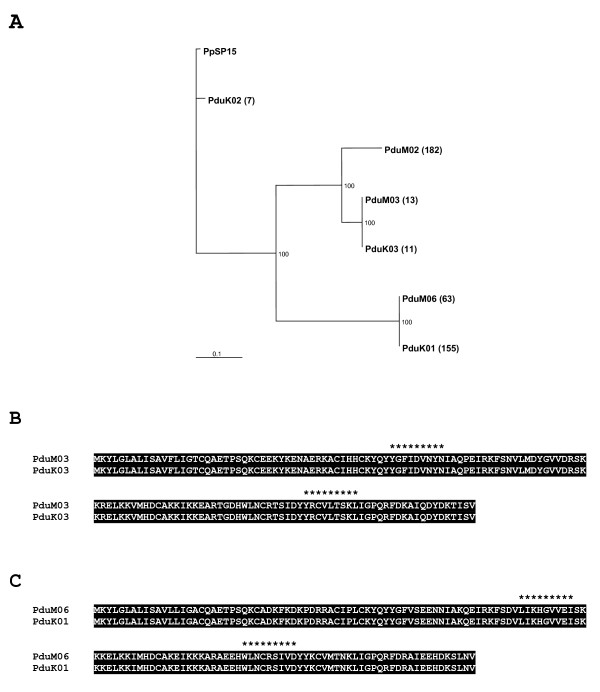
(A) Phylogenetic tree analysis of SP15-like proteins from *P. papatasi *(PpSP15), *P. duboscqi *Mali (PduM02, PduM03 and PduM06) and *P. duboscqi *Kenya (PduK02 and PduK03 and PduK01). (B) Sequence alignment of the orthologues PduM03 and PduK03. Black-shaded amino acids represent identical amino acids and * at the top of the amino acids denotes potential T cell epitopes as searched by using the TEPITOPE software. (C) Sequence alignment of the orthologues PduM06 and PduK01. Black-shaded amino acids represent identical amino acids * at the top of amino acids denotes potential T cell epitopes as searched by using the TEPITOPE software.

Immunisation of mice with PpSP15 protein or DNA plasmid coding for this protein from the saliva of *P. papatasi *was previously shown to protect mice against *L. major *infection [9]. Three members of the PpSP15-like family were identified in each sandfly cDNA library, PduM02, 03 and 06 from the Mali cDNA library and PduK01, 02 and 03 from the Kenya cDNA library. Phylogenetic tree analysis of these proteins resulted in the formation of three distinct groups (Figure 4A). Two groups with single members including PpSP15 and PduK02, a group with three members – including a cluster of orthologous sequences PduM03 and PduK03 – and the third group that includes the orthologues PduM06 and PduK01 (Figure 4A). In the third group, PduK01 is highly represented in this library (155 transcripts) as compared with PduM06 (63 transcripts), overall, these sequences contained more (PduK01) or fewer (PduM06) sequences from a particular value than expected from a random distribution, as evaluated by the χ^2 ^test, thus, this suggest that this protein is more frequent in the Kenya population than in the Mali population. When orthologues were compared, we observed a 100% identity between PduM03 and PduK03 (Figure 4B) and 100% identity between PduM06 and PduK01 (Figure 4C). Using TEPITOPE software on these sequences we identified two potential MHC class II T-cell epitopes in PduM03, YGFIDVNYN and YRCVLTSKL (Figure 3B). Two potential T-cell epitopes were found in PduM06, LIKHGVVEI AND WLNCRSIVD (Figure 4C).

### PpSP12-like proteins

**Figure 5:**
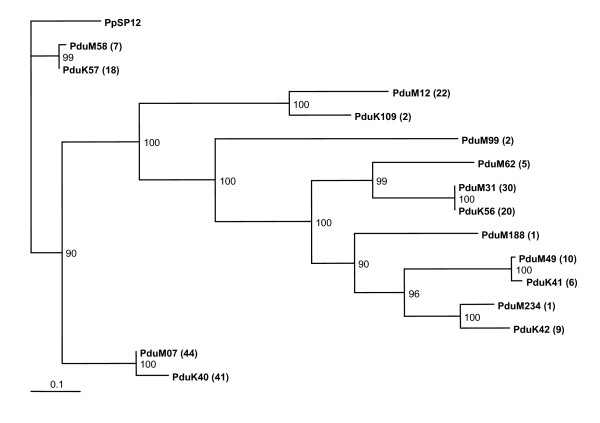
(A) Phylogenetic tree analysis of SP12 like proteins from *P. papatasi *(PpSP12), *P. duboscqi *Mali (PduM58, PduM12, PduM99, PduM62, PduM31, PduM188, PduM49, PduM234 and PduM07) and *P. duboscqi *Kenya (PduK57, PduK109, PduK56, PduK41 and PduK42).

**Figure 6:**
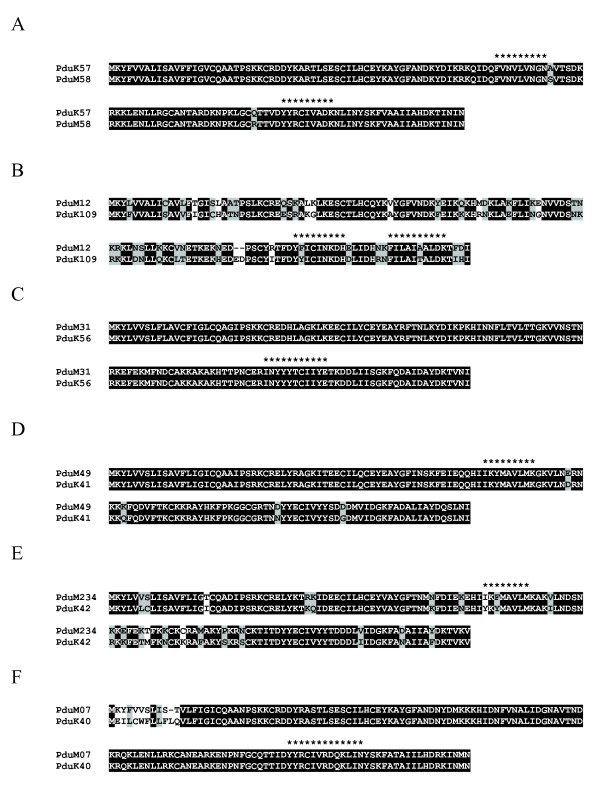
Sequence alignment of the orthologues (A) PduK57 and PduM58; (B) PduM12 and PduK109; (C) PduM31 and PduK56; (D) PduM49 and PduK41; (E) PduM234 and PduK42; (F) PduM07 and PduK40. Black-shaded amino acids represent identical amino acids, grey-shaded amino acids represent conserved amino acids and * at the top of amino acids denotes potential T cell epitopes as searched by using the TEPITOPE software.

This family of proteins was previously described in the salivary glands of *P. papatasi *and is a protein of 12 kDa with unknown function [9]. Transcripts with homologies to this protein were also found in the two sandfly cDNA libraries in the present work. Interestingly, this family of proteins had many members that were unlike the other salivary proteins in this sandfly in either location. For the Mali population we identified eight members, PduM07, 12, 31, 32, 49, 58, 62, and 99, and for the Kenya strain six members were identified, PduK40, 41, 42, 56, 57, and 109. Phylogenetic tree analysis resulted in the formation of 3 major clades (Figure 5), one clade containing PpSP12 and two orthologues PduM58 and PduK57, a second clade containing the orthologues PduM07 and PduK40 and a third clade containing a rapidly diverging salivary proteins, including various clusters of orthologous sequences such as PduM12 and PduK109, PduM31 and PduK56, PduM49 and PduK41, PduM234 and PduK42, and PduM07 and PduK40 (Figure 5). Sequence comparison between the different SP12-like orthologues resulted in a high level of identity among these proteins (Figure 6). PduM58 and PduK57 were 98.6 % identical and the predicted T-cell epitopes (Figure 6A) were 100% identical; PduM12 and PduK109 were 71.6% identical and the predicted T-cell epitopes were 89% identical (Figure 6B); PduM31 and PduK56 were 100 % identical (Figure 6C); PduM49 and PduK41 were 97% identical and the predicted T cell epitope was 100% identical (Figure 6D); PduM234 and PduK42 were 84.4 % identical and the predicted T cell epitope was 75% identical; PduM07 and PduK40 were 93% identical and the predicted T-cell epitope was 100% identical.

### D7-like proteins (SP28 and SP30)

**Figure 7:**
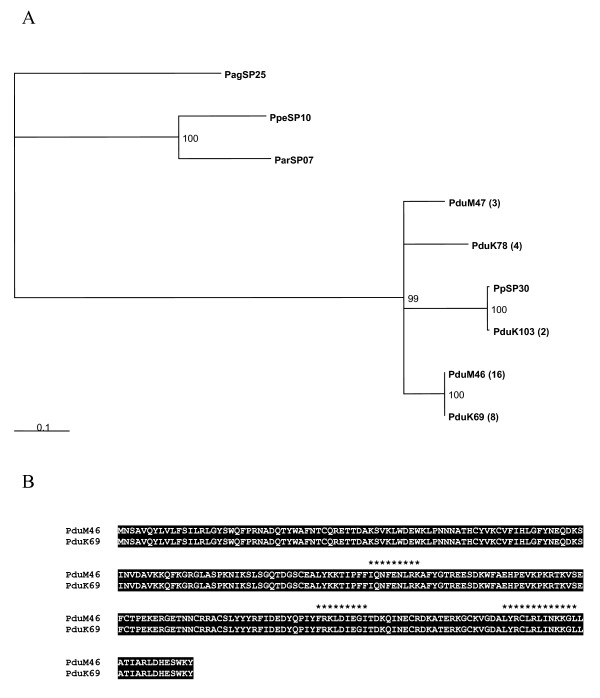
(A) Phylogenetic tree analysis of SP30 like (D7 like) proteins from *P. papatasi *(PpSP30), P. argentipes (PagSP25), P. perniciosus (PpeSP10), P. ariasi (ParSP07), *P. duboscqi *Mali (PduM47 and PduM46) and *P. duboscqi *Kenya (PduK78, PduK103 and PduK69). (B) Sequence alignment of the orthologues PduM46 and PduK69. Black-shaded amino acids represent identical amino acids and * at the top of the amino acids denotes potential T cell epitopes as searched by using the TEPITOPE software.

Transcripts with homology to the D7 family of proteins were identified in both cDNA libraries. D7 protein was previously reported in mosquitoes [17] and sandflies [18]. Only recently has its function been described from the saliva of Anopheles mosquito as a anti-clotting factor [19], and as a serotonin and small amine-binding protein [20]. Phylogenetic tree analysis of D7 proteins from various sandflies, including transcripts from Mali and Kenya, resulted in the formation of six different clades. Three clades are clusters of orthologous sequences that include PpeSP10 (*P. perniciosus *D7) and ParSP07 (*P. ariasi *D7), PpSP30 (*P. papatasi *D7) and PduK103 and a third cluster containing PduM46 and PduK69. Sequence comparison from the orthologues PduM46 and PduK69 showed 100% sequence identity and sharing of three potential T cell epitopes (Figure 7B).

### SP32-like proteins

**Figure 8:**
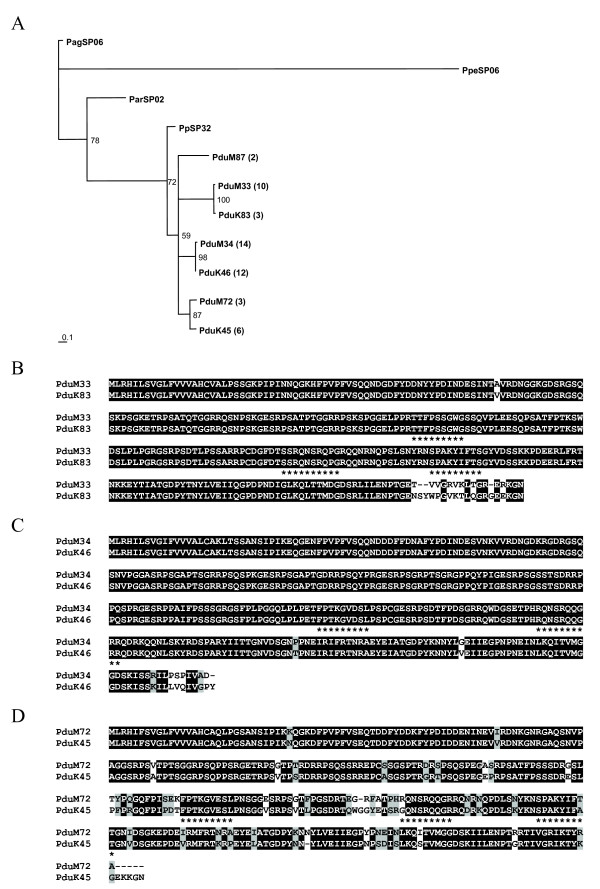
(A) Phylogenetic tree analysis of SP32-like proteins from *P. papatasi *(PpSP32), *P. argentipes *(PagSP06), *P. perniciosus *(PpeSP06), *P. ariasi *(ParSP02), *P. duboscqi *Mali (PduM87, PduM33, PduM34 and PduM72) and *P. duboscqi *Kenya (PduK83, PduK46 and PduK45). (B) Sequence alignment of the orthologues PduM33 and PduK83; (C) PduM34 and PduK46; (D) PduM72 and PduK45. Black-shaded amino acids represent identical amino acids and * at the top of the amino acids denotes potential T cell epitopes as searched by using the TEPITOPE software. (C) Sequence alignment of the orthologues PduM06 and PduK01. Black-shaded amino acids represent identical amino acids, grey-shaded amino acids represent conserved amino acids, * at the top of amino acids denotes potential T cell epitopes as searched by using the TEPITOPE software.

This protein family belongs to the silk-related and collagen-like protein in sandflies [9]. This type of protein has not been described in other blood feeding arthropods, yet it is present in the Phlebotomus as well as in the Lutzomyia sandflies [18]. These proteins are characterised by a large number of low complexity amino acids such as Glycine (G), arginine (R), proline (P) and serine (S), throughout the molecule (Figure 8). Phylogenetic tree analysis of various SP32-like proteins from different sandflies, including *P. duboscqi *Mali and Kenya, resulted in the formation of various clades (Figure 8A). Three of these clades are clusters of orthologues sequences: the first clade contains PduM33 and PduK83, the second clade contains PduM34 and PduK46, and the third clade contains PduM72 and PduK45 (Figure 8A). Sequence comparison between the SP32-like orthologues from Kenya and Mali resulted in a high degree of homology (Figure 8B,C,D). PduM33 and PduK83 had 96.1% sequence identity; additionally, two of the three potential T-cell epitopes (TTFPSSGWG AND SSRQNSRQPG) are 100 % identical (Figure 8B). PduM34 and PduK46 are 97.1 % identical and the two potential Tcell epitopes (FPTKGVDSL and RQNSRQQGRR) are 100% identical (Figure 8C). PduM72 and PduK45 are 84% identical, one potential T cell epitope (FPTKGVESL) is 100 % identical and the other two potential T cell epitopes (GQNSRQQRG and SPAKYIFAT) are 89% identical (Figure 8D).

### Antigen 5-related protein

**Figure 9:**
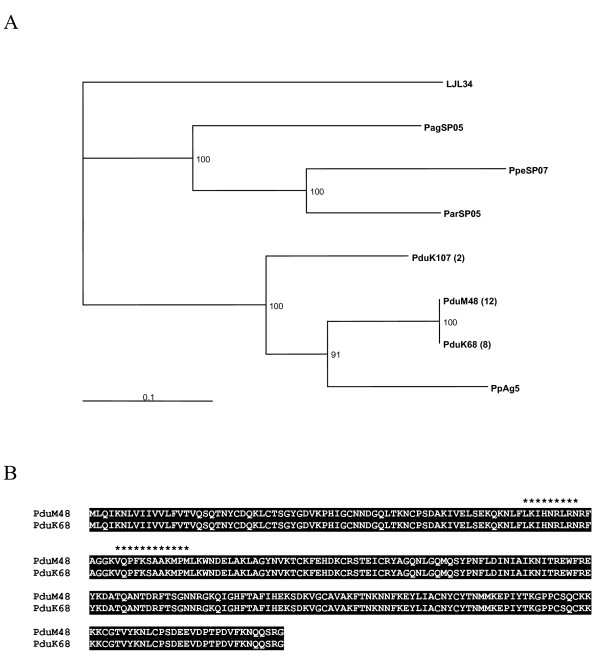
(A) Phylogenetic tree analysis of antigen 5-like proteins from *P. papatasi *(PpAg5), *P. argentipes *(PagSP05), *P. perniciosus *(PpeSP07), *P. ariasi *(ParSP05), *L. longipalpis *(LJL34), *P. duboscqi *Mali (PduM48) and *P. duboscqi *Kenya (PduK107 and PduK68). (B) Sequence alignment of the orthologues PduM48 and PduK68. Black-shaded amino acids represent identical amino acids and * at the top of the amino acids denotes potential T cell epitopes as searched by using the TEPITOPE software. (C)

This family of proteins belongs to the cysteine rich family of proteins (CRISP) found in wasp venom [21], hookworm [22], mosquitoes [23] and sandflies [18]. We found transcripts coding for this family of proteins in the *P. duboscqi *salivary gland cDNA libraries from Mali and Kenya. Phylogenetic tree analysis of antigen 5-related proteins from various sandflies, including the antigen 5-related proteins from *P. duboscqi *(Mali and Kenya), resulted in the formation of various clades – one of them containing the orthologues PduM48 and PduK68 from Mali and Kenya (Figure 9A). Sequence comparison of these orthologues resulted in 100% identity, including two potential T-cell epitopes (Figure 9B).

### Apyrase-like protein

**Figure 10:**
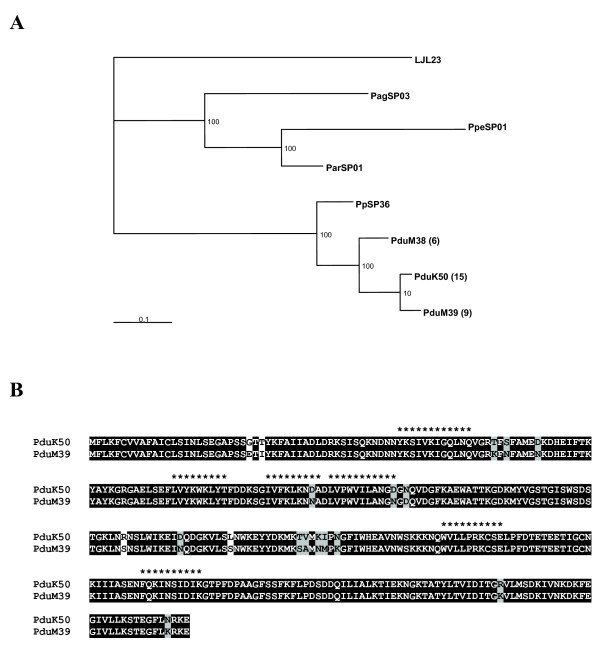
(A) Phylogenetic tree analysis of apyrase-like proteins from *P. papatasi *(PpSP36), *P. argentipes *(PagSP03), *P. perniciosus *(PpeSP01), *P. ariasi *(ParSP01), *L. longipalpis *(LJL23), *P. duboscqi *Mali (PduM38 and PduM39) and *P. duboscqi *Kenya (PduK50). (B) Sequence alignment of the orthologues PduK50 and PduM39. Black-shaded amino acids represent identical amino acids, grey-shaded amino acids represent conserved amino acids, and * at the top of the amino acids denotes potential T cell epitopes as searched by using the TEPITOPE software. (C)

Transcripts were found on the *P. duboscqi *Mali and Kenya cDNA libraries coding for a protein homologous to the *Cimex *family of apyrases [24], a protein also present in other organisms including sandflies [25], worms, mouse and humans [26,27]. Secreted apyrases function as potent anti-platelet factors by hydrolysing the platelet activator adenosine diphosphate (ADP). Phylogenetic tree analysis of apyrase-like proteins from different sandflies resulted in the identification of the apyrase-like orthologues PduK50 and PduM39 from the two cDNA salivary gland libraries (Figure 10A). The phylogenetic tree also shows that the apyrase-like proteins from *P. duboscqi *are closely related to *P. papatasi *apyrases and apart from other Phlebotomus and Lutzomyia apyrases. Sequence comparison between the Mali and Kenya orthologues shows a high degree of identity, 94.6%, between these two proteins (Figure 10B). Of interest, we observed five potential epitopes in this molecule, almost twice the number of epitopes identified from the other sandfly proteins. Four of these epitopes are 100% identical when comparing apyrase epitopes from Mali and Kenya proteins (Figure 10B).

### Yellow-related proteins

**Figure 11:**
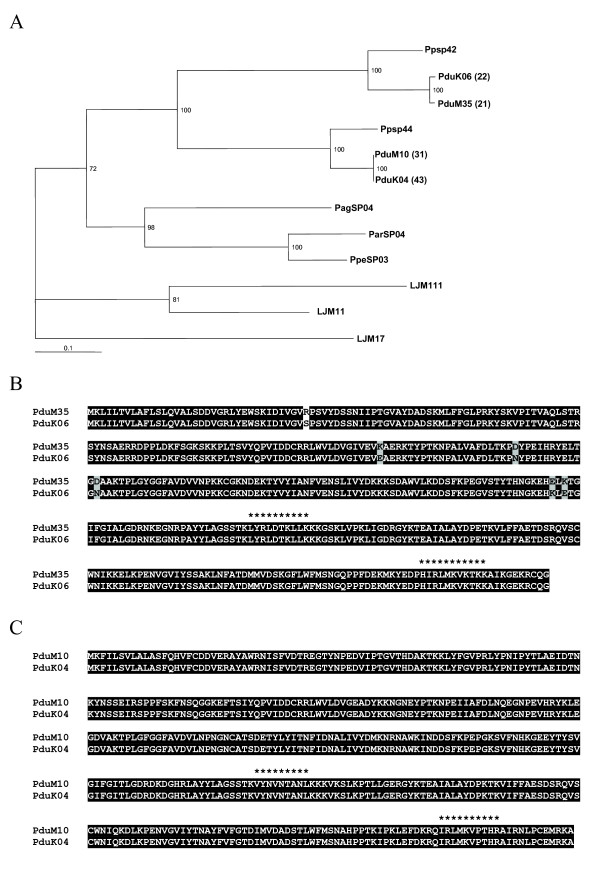
(A) Phylogenetic tree analysis of yellow-related proteins from *P. papatasi *(PpSP), *P. argentipes *(PagSP), *P. perniciosus *(PpeSP), *P. ariasi *(ParSP), *L. longipalpis *(LJ), *P. duboscqi *Mali (Pd) and *P. duboscqi *Kenya (PduK). (B) Sequence alignment of the orthologues PduK06 and PduM35; (C) PduK04 and PduM10. Black-shaded amino acids represent identical amino acids, grey-shaded amino acids represent conserved amino acids, and * at the top of the amino acids denotes potential T cell epitopes as searched by using the TEPITOPE software.

We identified in the *P. duboscqi *Mali cDNA library 2 transcripts (PduM10 and PduM35) and in the Kenya cDNA library 2 transcripts (PduK04 and PduK06) coding for a yellow related protein, a protein previously described in the saliva of *P. duboscqi *[28], other sand flies [18] and other insects[29]. Volf et al. [28] reported lectin activity of 42 kDa yellow-related protein purified from *P. duboscqi *lysates. However, the function of this protein in the saliva of insects remains unknown. Notably, a homologous protein was purified from *Aedes aegypti *midgut having a dopa decarboxylase activity [30]; this activity in the saliva of sandflies remains to be tested. Phylogenetic tree analysis of yellow-related proteins from different sandflies, including Mali and Kenya, resulted in the formation of five different clades (Figure 11A). Clusters of othologous sequences for the Mali and Kenya strain were found in the first two clusters, one containing the orthologues PduK06 and PduM35, and the other containing PduM10 and PduK04 (Figure 11A). Sequence comparison of PduK06 and PduM07 resulted in 97.5% identity (Figure 11B) and in 100% identity in the two potential T-cell epitopes identified (Figure 11B). Sequence comparison of PduM10 and PduK04 resulted in 100 % identity, including the two T-cell epitopes identified (Figure 11C).

## Conclusion

Salivary transcriptome and proteome analysis of *P. duboscqi *has resulted in a better understanding at the molecular level of the repertoire of proteins present in the saliva of this sandfly (Tables 1 and 2). Most salivary transcripts identified from the *P. duboscqi *cDNA libraries are very similar to those of the salivary proteins previously identified in *P. papatasi*. This is not surprising, because both *P. papatasi *and *P. duboscqi *belong to the same subgenus (Phlebotomus) and are proven natural vectors of *L. major*. A clear difference between *P. duboscqi *and *P. papatasi *cDNA libraries was the presence in *P. duboscqi *of an adenosine deaminase (the transcript and the protein). Adenosine deaminase has been reported in Aedes and Culex mosquitoes [31] and in the sandfly *L. longipalpis*; however, not in sandflies from the genus Phlebotomus [15].

This salivary transcriptome analysis allowed us to compare the salivary proteins of a sandfly from two different geographical locations. We investigated whether the salivary proteins from two different sites (Mali and Kenya) would be divergent, as previously reported with the salivary protein maxadilan when comparing *L. longipalpis *sandflies from Costa Rica, Colombia, and Brazil [12]. In the present work, we performed a global comparative analysis of the most abundant salivary proteins of sandflies from two locations, and searched for orthologues using phylogenetic analysis. We found the majority of the proteins to be highly conserved at both the aa and the nucleotide levels. We found that at least five families of proteins (SP15-like, SP12-like, D7-like, antigen 5-like, and yellow-related protein) were 100% identical in sandflies from Mali and Kenya. The other families were also highly conserved (94.6% to 99.8%) with the exception of three proteins that had moderate homology (of 18 orthologous sequences): two SP12-like members that were 84.4% and 71.6% identical, respectively, and a SP32-like member that was 84% identical.

Because cellular immune responses to sandfly saliva – particularly a DTH response – was previously associated with protection against Leishmania infection [5,9], we wanted first to identify potential MHC class II T-cell epitopes, which are required for DTH T cell-dependent responses and then determine whether these putative epitopes were also conserved among salivary proteins from these sandflies. The majority of potential T-cell epitopes were highly conserved among the different sandfly proteins; in fact, the majority of potential T cell epitopes were 100% identical, with the exception of only five epitopes that were 75% to 90% identical. These data suggest that even if the overall level of identity of some salivary proteins (Mali vs Kenya) is not 100%, the proteins have the potential to cross-react, at least at the level of cellular immune response (DTH) because of the high conservation of their T-cell epitopes that can be presented in the proper MHC class II context. This assumption needs to be tested experimentally.

A possible explanation for the conservation of salivary proteins include recent establishment of these sandflies in these regions with little or no evolutionary pressure from host immune response on these salivary proteins; or evolutionary pressure to keep these sequences constant (negative selection). Additionally, the location of these sand flies is more than 2000 thousand of kilometers apart. Then, it is difficult to suggest that there is a continuous exchange of sand flies in the whole sub-Saharan Africa moving from Kenya all the way to Mali or is also possible that the gene flow may be very low. In history, this area was affected by dramatic aridization (~5 millions years ago) [32] and consequent creation of Sahel as a unique transient formation (~3 milions years ago) [33], events that might led to separation and later rejoining of Eastern and Western populations of *P. duboscqi*. Further studies are needed to determine if these two populations are genetically isolated.

A DTH response to *P. papatasi *bites in mice was experimentally demonstrated to help these sandflies to probe and feed faster [34]. It was shown that this type of response considerably increased blood flow at the site of the bite (after subsequent sandfly challenge), creating a favorable environment for feeding. It is thus possible that this type of immune response may favor sandfly survival in nature and therefore will also favor the presence of highly conserved sequences in their salivary proteins.

The data presented in this work are in contrast to previous studies performed with the salivary protein maxadilan from the sandfly *L. longipalpis*, which was shown to be highly divergent between sandflies of distinct locations [12]. In contrast, PpSP15 from *P. papatasi *was shown to be highly conserved when comparing sandflies from different locations and isolates from field and laboratory colonies [35]. Therefore, it is possible that Phlebotomus salivary proteins are more conserved in general than proteins present in the saliva of Lutzomyia sandflies, perhaps due to the benefit accrued in increased feeding due to the host DTH response. It is also important to take into account that *L. longipalpis *is allegedly a complex of cryptic species [36], hence the larger variability observed in their salivary protein. Additionally, if *P. duboscqi *is a much older sand fly than *L. longipalpis*, it may be possible that Phlebotomus sand flies are more stable species which could explain the high conservancy of salivary proteins in the two different Phlebotomus species (*P. papatasi *and *P. duboscqi*).

Sandfly salivary components are potential vaccine candidates to control Leishmania infection. Our results suggest that *P. duboscqi *salivary protein that may be able to produce a protective cellular immune response should be able to induce the same immune response in hosts from distant geographical locations in the Sub-Saharan Africa where *P. duboscqi *is present.

## Methods

### Sandfly capture

Female *Phlebotomus duboscqi *sandflies were captured alive with solid-state miniature light traps(John Hock Company Ins., Gainsville, FL) and mouth aspirators in the villages of Kemena (-6° 54' 37", 13° 07, 22") Baraoueli Distric, Mali. The live flies were held in paper holding containers and stored in a cooler until they could be transported to the laboratory. In the laboratory, sandflies were identified to species using appropriate taxonomic keys for West Africa [37] and the salivary glands dissected and stored in groups of 20 pairs in RNA *later*^® ^solution (Ambion) and stored at 4°C until use.

### Sandfly salivary glands

Adult *Phlebotomus duboscqi *from a colony originated from Kenya were kept with free access to a 30% solution of sucrose. Salivary glands from recently emerged and 1- to 2-day-old adult female flies were dissected and transferred to 10 or 20 μl HEPES 10 mM pH 7.0, NaCl 0.15 M in 1.5 ml polypropylene vials, usually in groups of 10 pairs of glands in 20 μl of HEPES saline, or individually in 10 μl of HEPES saline. Salivary glands were stored at 75°C until needed.

### Salivary gland cDNA libraries

*Phlebotomus duboscqi *(Mali and Kenya) salivary gland mRNA was isolated from 45 and 55 salivarygland pairs, respectively, using the MicroFastTrack mRNA isolation kit (Invitrogen, SanDiego, CA). The PCR-based cDNA library was made following the instructions for the SMART cDNA library construction kit (BD-Clontech, Palo Alto, CA) with some modifications [14]. The obtained cDNA libraries (large, medium and small size) were plated by infecting log phase XL1-blue cells (Clontech) and the amount of recombinants was determined by PCR using vector primers flanking the inserted cDNA and visualised on a 1.1 % agarose gel with ethidium bromide (1.5 ug/ml).

### Massive sequencing of cDNA libraries

*P. duboscqi-*Mali and *P. duboscqi-*Kenya salivary gland cDNA libraries were sequenced as previously described using an Applied Biosystems 3730xl DNA Analyzer and a CEQ 2000XL DNA sequencing instrument (Beckman Coulter, Fullerton, CA) [18].

### Bioinformatics

Detailed description of the bioinformatic treatment of the data appear in [18,38,39]. Briefly, primer and vector sequences were removed from raw sequences and quality of sequence determined. Sequences were compared with the GenBank non-redundant (nr) protein database using the standalone Blastx program found in an executable package as previously described [40]. Related sequences were grouped into contigs and aligned using a CAP assembler. Contigs and singletons (contig containing only one sequence) were compared using the program blastX, blastN, or rpsBlast [40] to the non-redundant (nr) protein database of the National Center of Biological Information (NCBI), to the gene ontology database (GO) [41], the Conserved Domains Database (CDD) that includes all Pfam [42], SMART [43] and COG protein domains in the NCBI [44]. Additionally, contigs were compared with a customised subset of the NCBI nucleotide database containing either mitochondrial (mit-pla) or rRNA (rrna) sequences. Identification of putative secreted proteins was conducted using the SignalP server [45]. The three frame translation of each dataset was used to determine open reading frames (ORF). Only ORFs that started with a methionine and were longer than 40 amino acid (aa) residues were submitted to the SignalP server. The grouped and assembled sequences, BLAST results and signal peptide results were combined in an Excel spreadsheet and manually verified and annotated.

### Phylogenetic analysis

Protein families, identified through the bioinformatics analysis, were further analysed using phylogenetics. Consensus protein sequences of the identified protein families from each of the sandflies used in this analysis were compared with related sequences from sandfly vectors as well as non-sandfly species obtained from GenBank. Sequences were aligned using ClustalX [46] and manually refined using BioEdit sequence editing software [47]. Phylogenetic analysis was conducted on protein alignments using Tree Puzzle version 5.2 [48] incorporating the appropriate model of evolution defined by ProtTest [49]. Tree Puzzle constructs phylogenetic trees by maximum likelihood using quartet puzzling, automatically estimating internal branch node support (100,000 replications). Derived trees were visualised using TreeView [50].

### T-cell epitope prediction

The TEPITOPE software package [51] that searches for promiscuous HLA-class II binding peptides and human T-cell epitopes was set at threshold of 4% and run with the 25 different HLA-DR alleles. The promiscuous epitopes were selected from the *P. duboscqi *protein sequences tested that were predicted to bind at least 50% of the MHC class II molecules.

### SDS-PAGE

For *P. duboscqi *salivary glands, NuPAGE 10% Bis Tris gels (Invitrogen) were used. Gels were run with NuPAGE MES SDS running buffer (Invitrogen), according to the manufacturer's instructions. To estimate the molecular weight of the samples, SeeBlue™ markers from Invitrogen (myosin, BSA, glutamic dehydrogenase, alcohol dehydrogenase, carbonic anhydrase, myoglobin, lysozyme, aprotinin, and insulin, chain B) were used. The salivary gland homogenate was treated with equal parts of 2× SDS sample buffer (8% SDS in Tris-HCl buffer, 0.5 M, pH 6.8, 10% glycerol and 1% bromophenol blue dye). For aminoterminal sequencing of the salivary proteins, 35 homogenised pairs of salivary glands were electrophoresed and transferred to polyvinylidene difluoride (PVDF) membrane using NuPAGE transfer buffer, 10% methanol as the transfer buffer on a Blot-Module for the XCell II Mini-Cell (Invitrogen). The PVDF membrane was charged in 100% methanol for 30 seconds prior to the transfer on a Blot-Module for the XCell II Mini-Cell (Invitrogen). Upon transfer, the membrane was washed three times for five minutes with ultrapure water, and then treated for five minutes with a staining solution containing 0.025% Coomasie brilliant blue and 40% methanol in the absence of acetic acid. The membrane was partially destained in a solution of 50% methanol for ten minutes, then rinsed several times with ultrapure water. The membrane was allowed to dry before the stained bands were cut from the membrane and subjected to Edman degradation using a Procise sequencer (Perkin-Elmer Corp.)

To determine the cDNA sequences corresponding to the aa sequence obtained by Edman degradation, we used a search program that checked these aa sequences against the three possible protein translations of each cDNA sequence obtained in the DNA sequencing project. A more detailed account of this program is found elsewhere [14].

## Authors' contributions

HK constructed salivary gland cDNA library, carried out sequencing and proteome analysis and drafting of the manuscript; JMA carried out bioinformatic and comparative analysis, participated in sequence alignment and drafting of the manuscript; SK participated in design and coordination of the study, carried out sand fly identification in the filed and drafting of the manuscript; FO carried out phylogenetic analysis, sequence alignment and epitope analysis; PGL carried out entomological studies and drafting of the manuscript; VM carried out the sequence of sand fly transcripts; CSS coordinated entomological studies and identification of field specimens; SS carried out entomological studies, capture and identification of field specimens; IS carried out entomological studies, capture and identification of field specimens; MG carried out Edman-degradation of salivary proteins; LS constructed sand fly salivary gland cDNA library; PV participated in study design and coordination of study; SD coordinated entomological studies; JGV conceived the study and participated in its design coordination and drafting of the manuscript. All authors read and approved the final manuscript.

## Figures and Tables

**Table 1:** Salivary gland cDNA coding for secreted proteins from the sand fly *Phlebotomus duboscqi *from Mali

**Name**	**Cleavage position**	**Mature MW (kDa)**	**pI**	**No. of sequence in cluster**	**Best match to NR protein database**	**E value**	**% identity**	**Present in proteome**	**NCBI Gene Accession No**.
PduM02	20–21	14.44	9.15	182	15 kDa salivary protein (*P. papatasi*)	2.00E-59	71	Yes	DQ826514
PduM29	28–29	26.28	8.52	79	28 kDa salivary protein (*P. papatasi*)	1.00E-79	55	Yes	DQ826515
PduM06	20–21	14.40	9.27	63	15 kDa salivary protein (*P. papatasi*)	9.00E-54	65	Yes	DQ826516
PduM01	19–20	26.66	7.79	60	28 kDa salivary protein (*P. papatasi*)	7.00E-94	63	Yes	DQ826517
PduM07	20–21	13.97	8.81	44	12 kDa salivary protein (*P. papatasi*)	8.00E-54	72	Yes	DQ826518
PduM10	18–19	43.36	7.74	31	44 kDa salivary protein (*P. papatasi*)	0	87	Yes	DQ826519
PduM31	20–21	14.18	8.41	27	12 kDa salivary protein (*P. papatasi*)	3.00E-29	48	Yes	DQ826520
PduM12	20–21	13.98	8.76	22	12 kDa salivary protein (*P. papatasi*)	5.00E-31	47		DQ826521
PduM35	18–19	42.60	9.29	21	42 kDa salivary protein (*P. papatasi*)	0	83	Yes	DQ826522
PduM46	19–20	27.64	8.84	16	30 kDa salivary protein (*P. papatasi*)	1.00E-127	82	Yes	DQ826523
PduM34	23–24	31.75	9.98	14	32 kDa salivary protein (*P. papatasi*)	1.00E-66	47		
PduM03	20–21	14.50	9.04	13	15 kDa salivary protein (*P. papatasi*)	7.00E-59	71		DQ826524
PduM48	19–20	28.81	9.06	12	antigen 5-related protein (*Lu. longipalpis*)	1.00E-101	63		DQ826525
PduM49	20–21	14.20	8.42	10	12 kDa salivary protein (*P. papatasi*)	2.00E-31	47		DQ826526
PduM33	19–20	31.42	9.54	10	32 kDa salivary protein (*P. papatasi*)	4.00E-78	53		DQ834330
PduM39	21–22	35.23	9.03	9	salivary apyrase (*P. papatasi*)	1.00E-148	78	Yes	DQ834331
PduM50	20–21	14.58	8.23	8	14 kDa salivary protein (*P. papatasi*)	8.00E-54	70		DQ834332
PduM57	20–21	14.63	8.80	*7*	14 kDa salivary protein (*P. papatasi*)	2.00E-56	75		DQ834333
PduM58	20–21	13.70	9.35	7	12 kDa salivary protein (*P. papatasi*)	4.00E-64	82		DQ834334
PduM38	21–22	35.83	7.36	6	salivary apyrase (*P. papatasi*)	1.00E-148	85	Yes	DQ834335
PduM60)	22–23	14.37	8.44	5	14 kDa salivary protein (*P. papatasi*)	7.00E-54	73		DQ834336
PduM51	20–21	29.44	4.22	5	Sen1p (*Plasmodium yoelii yoelii*)	9.00E-05	25		
PduM62	20–21	14.23	7.60	5	12 kDa salivary protein (*P. papatasi*)	6.00E-26	46		DQ834337
PduM54	21–22	46.05	7.26	5	ENSANGP00000018673 (*Anopheles gambiae*)	8.00E-92	44		DQ834338
PduM04	23–24	34.13	5.06	4	32.4 kDa salivary protein (*Lu. longipalpis*)	6.00E-57	40		DQ834339
PduM05	28–29	33.91	10.05	4	32.4 kDa salivary protein (*Lu. longipalpis*)	5.00E-58	42	Yes	
PduM64	20–21	37.01	4.70	4	hypothetical protein L58460 (*Lactococcus lactis*)	5.00E-07	22		
PduM32	20–21	14.14	8.41	3	12 kDa salivary protein (*P. papatasi*)	2.00E-29	48	Yes	DQ834340
PduM47	19–20	27.60	8.75	3	30 kDa salivary protein (*P. papatasi*)	1.00E-124	81		DQ834341
PduM77	19–20	26.86	8.85	3	28 kDa salivary protein (*P. papatasi*)	7.00E-94	64		DQ834342
PduM78	16–17	16.34	5.62	3	unknown (*Culicoides sonorensis*)	4.00E-21	37		DQ834343
PduM80	26–27	2.78	12.7	3	2.7 kDa salivary protein *P. perniciosus*	.004	38		DQ835355
PduM72	17–18	31.04	8.07	3	32 kDa salivary protein (*P. papatasi*)	7.00E-65	49		DQ835356
PduM73	17–18	57.64	5.50	2	adenosine deaminase (*Lu. longipalpis*)	2.00E-84	60	Yes	DQ835357
PduM89	18–19	14.55	5.15	2	unknown (*Culicoides sonorensis*)	1.00E-25	50		DQ835358
PduM99	20–21	13.91	8.89	2	12 kDa salivary protein (*P. papatasi*)	1.00E-28	46		DQ835359
PduM82	20–21	17.70	5.36	2	CG7013-PA (*Drosophila melanogaster*)	1.00E-71	76		DQ835360
PduM87	19–20	27.87	9.18	2	32 kDa salivary protein (*P. papatasi*)	1.00E-44	44		DQ835361

**Table 2:** Salivary gland cDNA coding for secreted proteins from the sand fly *Phlebotomus duboscqi *from Kenya

**Name**	**Cleavage position**	**Mature MW (kDa)**	**pI**	**No. of sequence in cluster**	**Best match to NR protein database**	**E value**	**% identity**	**Present in proteome**	**NCBI Gene Accession No**.
PduK01	20–21	14.40	9.05	155	15 kDa salivary protein (*P. papatasi*)	1.00E-53	65	Yes	DQ835362
PduK34	19–20	26.38	8.52	139	28 kDa salivary protein (*P. papatasi*)	1.00E-79	55	Yes	DQ835363
PduK35	20–21	26.71	7.52	82	28 kDa salivary protein (*P. papatasi*)	4.00E-92	62	Yes	DQ835364
PduK04	18–19	43.36	7.74	43	44 kDa salivary protein (*P. papatasi*)	0	87	Yes	DQ835365
PduK40	21–22	13.97	8.81	41	12 kDa salivary protein (*P. papatasi*)	6.00E-54	72	Yes	DQ835366
PduK06	18–19	42.53	9.25	22	42 kDa salivary protein (*P. papatasi*)	0	84	Yes	DQ835367
PduK49	22–23	14.43	8.23	22	14 kDa salivary protein (*P. papatasi*)	2.00E-52	70		DQ835368
PduK56	20–21	14.15	8.41	20	12 kDa salivary protein (*P. papatasi*)	1.00E-25	47		DQ835369
PduK57	20–21	13.65	9.23	18	12 kDa salivary protein (*P. papatasi*)	8.00E-64	82	Yes	DQ835370
PduK50	21–22	35.76	8.65	15	*salivary apyrase *(*P. papatasi*)	1.00E-142	83	Yes	
PduK46	23–24	33.59	9.47	12	32 kDa salivary protein (*P. papatasi*)	2.00E-65	47		DQ835371
PduK03	20–21	14.50	9.04	11	15 kDa salivary protein (*P. papatasi*)	7.00E-59	71		DQ835372
PduK58	22–23	14.86	8.63	10	14 kDa salivary protein (*P. papatasi*)	5.00E-53	75		DQ835373
PduK42	20–21	14.44	8.58	9	12 kDa salivary protein (*P. papatasi*)	2.00E-32	51		DQ835374
PduK68	19–20	28.78	9.06	8	antigen 5-related protein (*Lu. longipalpis*)	1.00E-101	63		DQ835375
PduK69	19–20	27.64	8.84	8	30 kDa salivary protein (*P. papatasi*)	1.00E-127	82	Yes	DQ835376
PduK02	20–21	14.46	9.19	7	15 kDa salivary protein (*P. papatasi*)	1.00E-76	96		DQ835377
PduK41	20–21	14.13	8.60	6	12 kDa salivary protein (*P. papatasi*)	4.00E-30	46		DQ835378
PduK45	26–27	27.02	9.38	6	32 kDa salivary-protein (*P. papatasi*)	1.00E-79	53		DQ835379
PduK77	27–28	2.53	12.41	5	No matches found				DQ835380
PduK70	21–22	34.80	9.13	4	32.4 kDa salivary protein (*Lu. Iongipalpis*)	3.00E-51	38	Yes	
PduK78	22–23	27.47	8.66	4	30 kDa salivary protein (*P. papatasi*)	1.00E-113	78		DQ835381
PduK74	21–22	36.07	7.66	3	ENSANGP00000018673 (*Anopheles gambiae*)	4.00E-86	45		
PduK83	24–25	31.36	9.19	3	32 kDa salivary protein (*P. papatasi*)	1.00E-79	54		DQ835382
PduK86	17–18	40.45	6.63	3	42 kDa salivary protein (*P. papatasi*)	1.00E-79	45		
PduK05	18–19	42.21	8.78	2	44 kDa salivary protein (*P. papatasi*)	0	98	Yes	
PduK60	17–18	57.28	6.15	2	adenosine deaminase (*Lu. longipalpis*)	5.00E-91	60	Yes	
PduK84	20–21	27.47	4.28	2	Sen1p (*Plasmodium yoelii yoelii*)	5.00E-05	25		DQ835384
PduK103	19–20	27.74	9.11	2	30 kDa salivary protein (*P. papatasi*)	1.00E-146	99		DQ835385
PduK107	19–20	28.91	9.22	2	antigen 5-related protein (*Lu. longipalpis*)	1.00E-99	64		DQ835386
PduK109	20–21	14.21	8.23	2	12 kDa salivary protein (*P. papatasi*)	3.00E-35	52		DQ835387
PduK110	19–20	16.53	5.94	2	16.1 kDa salivary protein (*Lu. longipalpis*)	2.00E-71	82		DQ835388
